# Nutrient sensing, growth and senescence

**DOI:** 10.1111/febs.14400

**Published:** 2018-02-15

**Authors:** Bernadette Carroll, Viktor I. Korolchuk

**Affiliations:** ^1^ Institute for Cell and Molecular Biosciences Newcastle University UK

**Keywords:** ageing, autophagy, growth, membrane potential, mTORC1, primary cilia, senescence

## Abstract

Cell growth is dictated by a wide range of mitogenic signals, the amplitude and relative contribution of which vary throughout development, differentiation and in a tissue‐specific manner. The ability to sense and appropriately respond to changes in mitogens is fundamental to control cell growth, and reduced responsiveness of nutrient sensing pathways is widely associated with human disease and ageing. Cellular senescence is an important tumour suppressor mechanism that is characterised by an irreversible exit from the cell cycle in response to replicative exhaustion or excessive DNA damage. Despite the fact that senescent cells can no longer divide, they remain metabolically active and display a range of pro‐growth phenotypes that are supported in part by the mTORC1‐autophagy signalling axis. As our understanding of the basic mechanisms of controlling mTORC1‐autophagy activity and cell growth continues to expand, we are able to explore how changes in nutrient sensing contribute to the acquisition and maintenance of cellular senescence. Furthermore, while the protective effect of senescence to limit cellular transformation is clear, more recently, the age‐related accumulation of these pro‐inflammatory senescent cells has been shown to contribute to a decline in organismal fitness. We will further discuss whether dysregulation of nutrient sensing pathways can be targeted to promote senescent cell death which would have important implications for healthy ageing.

**Abbreviations**
ADP: adenosine diphosphate
AMP: adenosine monophosphate
AMPK: AMP‐activated protein kinase
Atg5: autophagy related 5
Atg7: autophagy related 7
ATM: ataxia‐telangiectasia‐mutated
ATP: adenosine triphosphate
ATR: ataxia telangiectasia and Rad3‐related
BAG: Bcl‐2‐associated athanogene
CP110: centriolar coiled‐coil protein 110
DDR: DNA damage response
EGF‐R: epidermal growth factor receptor
EIF4EBP1 (4E‐BP1): eukaryotic translation initiation factor 4E binding protein 1
GAP: GTPase activating protein
GATA4: GATA binding protein 4
GEF: nucleotide exchange factor
*GLI2*: GLI family zinc finger 2
*GLIS2*: GLI‐similar 2
GTP: guanosine triphosphate
Hh: Hedgehog
HIF1a: hypoxia‐inducible factor 1‐alpha
HMEC: human mammary epithelial cells
hTERT: human telomerase reverse transcriptase
IFT20: intraflagellar transport protein 20
IFT88: intraflagellar transport protein 88
IGF‐R: insulin‐like growth factor 1 receptor
IHH: Indian hedgehog
IL1A: interleukin 1A
IL6: interleukin 6
INPP5E: inositol polyphosphate 5‐phosphatase
Kif3a: kinesin family member 3A
LKB1: liver kinase B1 (also known as serine/threonine kinase 11 (STK11)
MiT/TFE: microphthalmia/transcription factor E family
MK2/MAPKAPK2: MAP kinase‐activated protein kinase 2
mTORC1: mammalian target of rapamycin complex 1
NADH: nicotinamide adenine dinucleotide
NF‐kB: nuclear factor kappa‐light‐chain‐enhancer of activated B cells
NPH: nephronophthisis
OFD1: centriole and centriolar satellite protein
OIS: oncogene‐induced senescence
OP: oligodendrocyte progenitor
PC: primary cilia
PDGF‐R: platelet‐derived growth factor receptors
PGC1β: peroxisome proliferator‐activated receptor‐gamma coactivator‐1beta
PI3K: phosphatidylinositol‐4,5‐bisphosphate 3‐kinase
PIKK: phosphatidyl inositol 3′ kinase‐related kinases
PINK1: PTEN induced putative kinase 1
Rheb: Ras homologue enriched in brain
ROS: reactive oxygen species
RPE: retinal pigment epithelium
RTK: receptor tyrosine kinase
S6: ribosomal S6 kinase
SASP: senescence‐associated secretory phenotype
Sen‐β‐Gal: senescence‐associated beta‐galactosidase
SHH: sonic hedgehog
TASCC: TOR‐autophagy spatial coupling compartment
TSC: tuberous sclerosis complex
ZFP36L1: ZFP36 ring finger protein like 1

## Introduction

Growth needs to be tightly coordinated with the presence of nutrients and extracellular growth promoting cues at the cellular, tissue and organismal level. Unsurprisingly, perturbations in nutrient and growth factor sensing are tightly linked with human pathologies, particularly ageing and age‐related disease such as cancer, neurodegeneration and metabolic syndrome 1, 2. Understanding the basic mechanisms that maintain nutrient homeostasis in a healthy state and importantly, exploring how they are perturbed in pathologies is a thriving area of biomedical sciences. At the very heart of cell growth regulation lies the interplay between the mammalian target of rapamycin complex 1 (mTORC1), which promotes cellular anabolism, and the catabolic autophagy pathway.

Mammalian target of rapamycin complex 1 is a signalling hub which receives and integrates multiple inputs including the presence of growth factors, oncogenes, energy in the form of ATP, oxygen, reactive oxygen species and intracellular amino acids. Sensing of these factors is facilitated by an array of dedicated proteins which encompass the mTORC1 signalling network and which have been described in detail in a number of excellent reviews elsewhere 2, 3, 4, 5, 6, 7. Briefly, mitogenic signals serve ultimately to control the subcellular localisation and therefore activity of mTORC1 and its regulatory proteins. Growth factor‐activated receptors, cellular energy levels and amino acids such as arginine control mTORC1 by regulating the interaction of its master activator, the small GTPase Rheb (Ras homologue enriched in brain) with a negative regulator, tuberous sclerosis complex (TSC) 5, 8, 9, 10, 11, 12. In the absence of these factors, the TSC complex is recruited to the surface of the lysosome where the GTPase activating protein (GAP), TSC2 within the complex binds to and facilitates GTP hydrolysis of Rheb 9, 10, 11, 12 as well as physically sheltering Rheb from mTORC1 12.

To become activated by Rheb, mTORC1 needs to be recruited to the lysosomal surface by the family of Rag small GTPases which are present in a heterodimeric complex consisting of Rag A or B in complex with Rag C or D 2, 6. Unlike Rheb, Rag GTPases are cytoplasmic proteins which are tethered to the lysosome by another protein complex, the Ragulator, which further facilitates activation by GTP loading of RagA/B in its capacity as a nucleotide exchange factor (GEF) 2, 6. The amino acids arginine, leucine and glutamine are sensed by several molecular mechanisms all converging on and changing GTP/GDP binding status of the Rag heterodimer 6. The presence of these amino acids allows Rheb to interact with Rag‐bound mTORC1 which results in its activation although the underlying molecular mechanism remains unknown. It has been proposed that following its activation, mTORC1 can be released from the lysosome which allows it to phosphorylate its targets including regulators of protein translation machinery p70‐S6 kinase and 4E‐BP1 13. Active mTORC1 also suppresses the catabolic autophagy pathway, a process whereby intracellular components including damaged or surplus proteins, protein aggregates, organelles and pathogens are engulfed by a membrane to form vesicles called autophagosomes. These eventually fuse with the lysosome where their content is degraded and the constituents are released back into cytoplasm to serve in biosynthetic processes 3.

## mTORC1 in senescence

**Figure 1:**
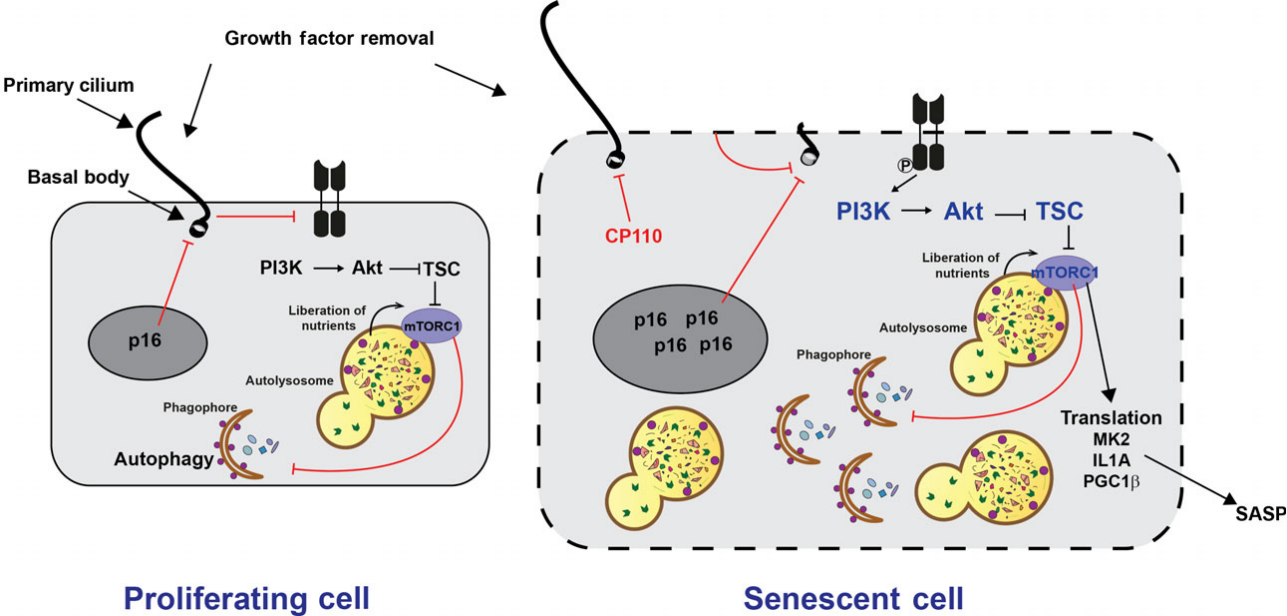
Dysregulation of growth factor sensing to mTORC1 and autophagy in senescence. In proliferating cells, growth factor removal induces cilia growth which downregulates growth factor signalling, mTORC1 and expression of cell cycle inhibitor p16. In senescent cells, cilia formation is abnormal (lack of elongation or increased length) which is a result of plasma membrane hypopolarisation, p16 or reduced CP110 (negative regulator of cilium growth) expression. As a result, PI3K/Akt signalling to mTORC1 is persistent and starvation fails to induce autophagy. Persistent growth factor and mTORC1 signalling may contribute to SASP. Red text indicates proteins/complexes that are downregulated in senescence; Blue text indicates that the activity of protein/complexes is upregulated in senescence.

In normal cells, the removal of mitogenic cues such as growth factors and amino acids inactivates mTORC1, slowing cell growth and activating autophagy to liberate nutrients and support cell survival. However, in some human disorders, mTORC1 does not respond appropriately to nutritional cues which contribute to pathological phenotypes. This can be exemplified using cellular senescence where mTORC1 becomes resistant to nutrient starvation 14, 15 and rather, the kinase complex is constitutively active (Fig. 1). This has been associated with defects in both growth factor and amino acid sensing pathways and in this review we will discuss the mechanisms that contribute to dysregulation of nutrient sensing and explore the repercussions for our understanding of cell growth regulation in health and disease.

Cellular senescence is a term to describe cells that have irreversibly exited the cell cycle. Senescence is classically considered to be a tumour suppressor mechanism that prevents potential cellular transformation upon oncogene activation or excessive DNA damage. At the same time, the age‐related accumulation of senescent cells has been implicated as an important contributor to age‐related decline in tissue and organ fitness 16. Cellular senescence was first described by Leonard Hayflick upon the observation that primary human diploid fibroblasts in culture can only divide a finite number of times before exiting the cell cycle 17. Subsequent research has implicated that telomere shortening is an important inducer of cell senescence, as well as excessive DNA damage from irradiation or following the replicative burst associated with oncogene activation 16, 18, 19, 20, 21. Activation of the DNA damage response (DDR) via PIKK family members, ATM and ATR triggers a signalling cascade including activation of p53 and the accumulation of cyclin‐dependent kinase inhibitors such as p16(INK4A), p19(ARF) and p21(WAF1/CIP1) 16, 20, 21, 22. Despite the fact that they can no longer divide, senescent cells are extremely metabolically active and are fundamentally characterised by many pro‐growth phenotypes such as an enlarged size, increased organelle content (including mitochondria and lysosomes), increased metabolism and the potent secretion of inflammatory mediators 16, 23, 24, 25, 26, 27. This latter phenotype is referred to as senescence‐associated secretory phenotype (SASP) and is associated with increased expression of cytokines and chemokines such as IL6 and IL8 27.

The activity of mTORC1 has been widely implicated in driving many of these phenotypes and indeed the observed insensitivity of mTORC1 to changes in the balance of mitogenic cues is likely to have wide implications for protein translation and cell metabolism. For example, mTORC1 can support mitochondrial biogenesis and SASP via protein translation‐dependent mechanisms. Specifically, mTORC1 regulates translation of MK2/MAPKAPK2 which controls senescence via phosphorylation of the RNA‐binding protein, ZFP36L1 preventing its binding to and degrading SASP RNA transcripts 28. Furthermore, mTORC1‐dependent translation of IL1A promotes transcription of SASP factors such as IL6 via NF‐kB 29. The mTORC1‐dependent biogenesis of mitochondria via PCG1β and the subsequent increase in intracellular ROS is also important for activating senescence‐associated DDR and ultimately SASP 30 (See Fig. 1). All of these reports use the mTORC1 inhibitor, rapamycin to demonstrate the specific role for mTORC1 in controlling SASP. Furthermore, rapamycin has been comprehensively shown to be able to slow the induction of senescence downstream of inducible p21, oxidative stress 31, DNA damage 32, oncogene activation 33 and replicative exhaustion 33, 34, demonstrating the central importance of mTORC1 to acquisition of senescence and driving senescence‐associated phenotypes. Furthermore, inhibition of growth factor signalling upstream of mTORC1 also slows senescence acquisition 35.

## Autophagy in senescence

**Figure 2:**
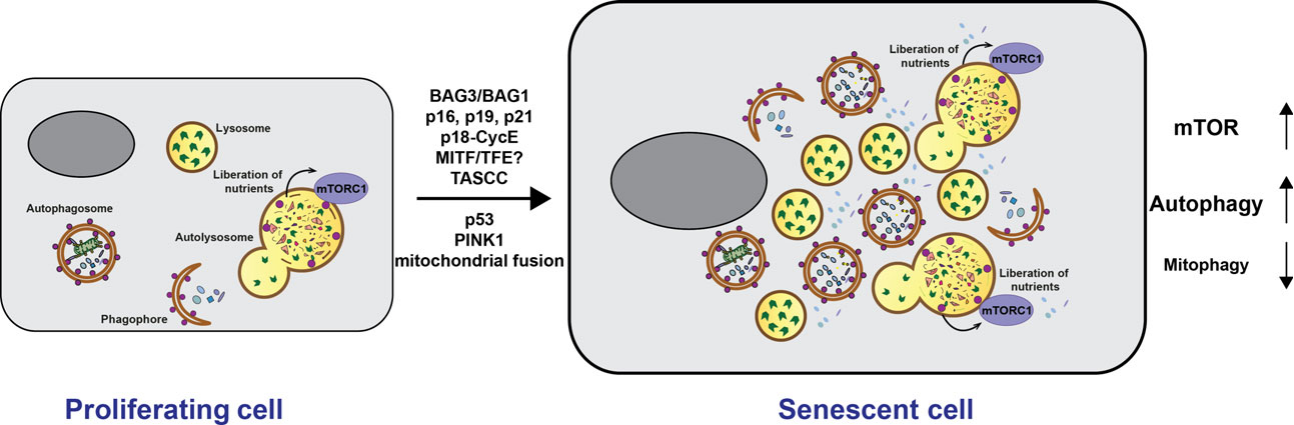
Senescence‐associated changes in the autophagosome–lysosome pathway. Senescent cells are characterised by increased numbers of autophagosomes and lysosomes, increasing flux through the pathway. This increases the generation of intracellular amino acids and supports activity of mTOR pathway. At the same time, selective autophagy pathways such as mitophagy may be suppressed in senescence. Some of the factors potentially leading to these changes in senescence are indicated. See text for further details.

Interestingly, in senescent cells, autophagy may become partially uncoupled from mTORC1 signalling and its inhibitory effect 14. We have demonstrated that autophagy flux, which reflects the activity of this degradative system, is significantly increased in senescent fibroblasts even in the presence of nutrients. This correlates with increased levels of intracellular amino acids both in fed and starved senescent cells. At the same time, autophagy is not further upregulated when cells are starved, consistently with the lack of mTORC1 inactivation in these conditions. Together with a well‐documented expansion of the lysosomal compartment, which is exploited in the Sen‐β‐Gal labelling of senescent cells, these data suggest that the entire autophagosome–lysosome system is upregulated in senescence (Fig. 2). Indeed, several studies have shown that autophagy is important for the maintenance of senescence phenotypes and knockdown of the essential autophagy genes *Atg5* or *Atg7* reduces SASP and can bypass senescence 36, 37.

A number of potential mechanisms can account for the increased autophagy in senescent cells. Autophagy has been suggested to increase in replicative senescence due to the change in the expression ratio of BAG (Bcl‐2‐associated athanogene) family of proteins. Specifically, an increase in the BAG3/BAG1 ratio in senescent cells may shift the balance of cellular proteolytic systems from proteasomal to autophagic degradation 38. Alternatively, expression of p16(INK4A), p19(ARF) or p21(WAF1/CIP1) not only induces senescence but also autophagy 39. Furthermore, a proteolytic Cyclin E fragment (p18‐CycE) was shown to facilitate DNA‐damage‐induced senescence and autophagy induction 40. The simultaneous mTORC1 and autophagy activation in senescence can be mediated by a specialised intracellular structure called the TOR‐autophagy spatial coupling compartment (TASCC) which is found in some forms of senescence, particularly induced by oncogene expression 36. TASCC was shown to allow compartmentalised generation of amino acids and other metabolites by autophagy and simultaneously stimulate mTORC1 signalling which promotes increased production of SASP factors, thus facilitating acquisition of senescence phenotypes. An MIT/TFE transcription factor‐dependent mechanism of simultaneous upregulation of autophagosome–lysosome pathway and mTORC1 has also recently been described in certain cancer models 41. Here, increased levels of MIT/TFE not only induce lysosomal biogenesis, but also expression of RagD which facilitates recruitment of mTORC1 to the lysosome and its activation. Although we did not observe differences in the expression of Rag GTPases in stress‐induced senescence (unpublished observations), this mechanism may be potentially responsible for uncoupling of autophagosome–lysosome pathway from the control by mTORC1 in other types of senescence. Thus, different mechanisms, some of which are still to be discovered, could be responsible for the senescence‐associated dysregulation of mTORC1/autophagy axis. It is important to note that intracellular amino acid concentrations in senescent cells are elevated only when normalised to the cell number which is a common normalisation method 14. However, senescent cells are grossly enlarged compared to control cells and when normalised by protein concentration, amino acid concentrations are comparable to controls 14. Although it could be envisaged that localised production of amino acids produced by hyperactive lysosomes could be responsible for the persistent mTORC1 in senescent cells, one should bear these unique problems in mind when analysing observations obtained in senescent cells.

The issue is further confused by the fact that autophagy is also suggested to prevent cellular senescence. Thus, it was shown that ROS can lead to perturbation of autophagic flux in senescent cells and restoration of flux can be achieved by AMPK activation 42, 43, 44. These conclusions seem to be consistent with the discussed above and well‐documented suppression of senescence by mTORC1 inhibitors which may, at least in part, act by autophagy upregulation 30, 44, 45. Similarly, recent reports suggest that autophagy can allow the bypass of RAS‐induced senescence and facilitate tumour growth 46. Several potential arguments can be offered to explain the contradictory role of autophagy in senescence. For example, inhibition of autophagy in young cells may result in senescence whilst secondary upregulation of autophagy in senescent cells may serve to maintain senescence phenotypes such as increased metabolic activity. Additionally, autophagy may differentially impact on the process of senescence acquisition depending on the relative expression of specific autophagy substrates. For example, it has been suggested that selective autophagy may prevent senescence by degrading a pro‐senescence factor GATA4 whilst senescence may indeed be facilitated by bulk autophagy through the TASCC 36, 47.

This uncoupling of bulk and selective forms of autophagy may be a general feature of senescence. Selective autophagic degradation of dysfunctional mitochondria, which is referred to as mitophagy is suggested to decline in senescent cells independent of the changes in the autophagosome–lysosome pathway 48, 49. Several mechanisms may account for this phenomenon. For example, cytoplasmic p53 upregulated in senescent cells can interact with Parkin, an ubiquitin ligase which tags defective mitochondria with ubiquitin for degradation, and prevents its translocation to mitochondria 50. Similarly, expression of mitophagy‐promoting protein kinase PINK1 becomes reduced with aging, suppressing mitophagy and enhancing the probability of senescence transition 51, 52. Finally, it has been observed that in senescent cells the mitochondrial network becomes hyperfused which prevents damaged mitochondria being engulfed by autophagosomes 48. Overall, an accumulation of dysfunctional mitochondria as a result of mitophagy impairment may lead to an increase in intracellular ROS and contribute to the senescence phenotype as has been recently demonstrated for muscle stellate cells. These cells become defective in proliferation and differentiation with age and eventually become senescent. At least part of this process is mediated by defective mitophagy which promotes oxidative stress, de‐repression of p16(INK4A) and ultimately senescence 49, 53.

## Primary cilia in cell growth and senescence

The unresponsiveness of mTORC1/autophagy to serum starvation in senescence is associated with a number of mechanisms including changes in membrane potential which impacts on the formation of specialised immobile membrane projections called primary cilia (PC). Defects in PC formation supports elevated and persistent PI3K/Akt signalling and loss of TSC2 recruitment to the lysosome which in turn renders mTORC1 and autophagy insensitive to growth factor withdrawal 14. Work by others indicates that the interaction between PC, PC‐associated signalling, cell growth and senescence appear to be very closely related (Fig. 1).

PC can be formed by almost all cells and function to ‘sense’ the extracellular environment, both physical and chemical signals, to control cell proliferation, differentiation and renewal. There is increasing appreciation for the role of PC in sensing and integrating mitogenic signals with wide implications for both basic cell biology and disease. PC are formed by a characteristic 9 + 0 microtubule arrangement, that is, they lack the central microtubule doublet that would support motility of cilia 54. They emanate from a basal body which is derived from a centrosome and thus the growth of PC is commonly associated with cells in a quiescent or non‐proliferating state. In fact, PC formation and cell proliferation were classically considered to be mutually exclusive and evidence suggests that PC function to inhibit or reduce cell growth. Defects in PC formation or function therefore have obvious repercussions for cancer and tumour suppressor mechanisms such as senescence.

Similar to senescent cells, mTORC1 in PC‐deficient cells is also resistant to serum starvation 14. Furthermore, some ciliopathies, the term used to describe a diverse group of genetic diseases that arise from PC defects are associated with increased mTORC1 55, 56 and increased senescence 57. Polycystic kidney disease which is characterised by cyst formation is associated with elevated mTORC1 activity and increased cell size 55. This study demonstrated that in control mice and cells, PC‐dependent sensing of fluid flow in the kidney promotes recruitment of the tumour suppressor LKB1 to cilia which leads to increased activation of AMPK in the basal body and an inhibition of mTORC1 55. In models defective in PC formation (e.g. knockdown of *Kif3a* and *IFT88*), mTORC1 activity is enhanced and both *in vivo* and *in vitro*, inducible knockdown of *Kif3a* results in increased cell size. Furthermore, the ciliopathy nephronophthisis (NPH) causes kidney failure in children and young adults and is associated with increased senescence as indicated by increased p16 expression and Sen‐β‐Gal 57. In this specific model, senescence is associated with loss of function of the transcription factor, GLIS2 (GLI‐similar 2), which is regulated by Hedgehog (Hh) signalling pathway (the most well‐studied pathway downstream of PC). Consistent with this study, the Hh‐responsive transcription factor, GLI2 which can bind to the p16 promoter and inhibit its expression 58 is downregulated in replicative senescent cells 59 and in stress‐induced senescent cells (unpublished data). Thus, loss of GLI2 and the subsequent failure to repress p16 expression may be a universal contributor to the senescence phenotype.

At the same time, p16 inhibits the formation of PC in human mammary epithelial cells (HMEC) 58, suggesting that perhaps this mechanism could also serve to reinforce the pro‐growth phenotypes in senescence. Why or how the levels of GLI2 are reduced in senescence, however, is unknown. Equally, how this transcription factor may impact on p21‐mediated senescence or in oncogene‐induced senescence (OIS) remains to be seen. Although these reports indicate senescent 14 and p16 + 58 cells fail to form cilia, another report by Breslin *et al*. (2014) show increased frequency and length of PC in replicative senescent human fibroblasts due to loss of the PC‐inhibitory protein, CP110 59. While these disparities may be due to differences in senescence induction, cell line or culture conditions, they may equally indicate that a fine balance in PC formation, length and turnover is required for their proper functioning and downstream signal transduction. For example, very few ciliopathies are associated with a complete loss of cilia and human disease is characterised by a very diverse array of phenotypes.

## Cell growth and membrane integrity

As we have discussed above, defects in PC elongation have been associated with p16 expression 58, loss of CP110 59 and we have recently shown that hypopolarisation of cell plasma membrane can perturb cilia growth 14 (Fig. 1). Membrane potential shows considerable variation and is intimately linked to cell type, function and degree of differentiation. Proliferating fibroblasts have a resting membrane potential of ~ 20 mV, this decreases to ~ 70 mV in non‐proliferating/quiescent fibroblasts and our data indicate that this hyperpolarisation is lost in senescence. Proper control of membrane potential is mediated by K+ channels and is required for cell growth, proliferation and the proper balance of other intracellular ions such as calcium. Numerous studies, in many model systems indicate that K+ channel blockers inhibit cell proliferation and that while cells in G0/G1 phase are more depolarised, hyperpolarisation of the membrane is required for the transition from G1 to S phase 60. Consistent with this, changes in the potential of the plasma membrane may play an influential role in senescence acquisition as membrane hyperpolarisation (by activating K+ channels using pinacidil) prolongs proliferation of primary fibroblasts and delays the onset of senescence.

CDK inhibitors contribute to cell cycle arrest and senescence to differing degrees in different cell types; for example, p27 is important in oligodendrocyte progenitor (OP) cells 61 whilst melanocytes show preferential sensitivity to p16 expression 62. Depolarisation‐associated cell cycle arrest of OP cells was shown to be dependent on p21 and p27 expression but not on p16 or p19 61. Whether plasma membrane hypopolarisation contributes to cell cycle arrest in cell types where senescence is dependent on p16 or p19 is currently unknown. Moreover, there is little understanding about the extent to which these cell‐type‐specific mechanisms of cell cycle regulation contribute to senescence acquisition or senescence phenotypes, nor whether there is any significance to the fact that senescent cells are arrested in both G1 and G2 phases 63, 64.

Our data indicate that defects in membrane potential perturb PC formation and that hyperpolarisation of the senescent cell membrane promotes elongation of cilia which is sufficient to reduce activity of PI3K/Akt pathway (in a cilia‐dependent manner) and restores sensitivity of mTORC1 in senescent cells to serum starvation. At present, it is not clear mechanistically how cilia can directly impact on growth factor signalling. On one hand, growth factor receptors such as PDGF‐R, EGF‐R and IGF‐R have been shown to localise and potentially activated in the ciliary axoneme or basal region 65, 66, 67. At the same time, negative regulators of growth factors signalling, such as the inositol polyphosphate 5‐phosphatase, INPP5E are recruited to PC. Mutations in INPP5E have been shown to contribute to polycystic kidney disease as a result of elevated PI3K/Akt and mTORC1 signalling 68. It is possible that recruitment of such negative regulators to the plasma membrane and PC are perturbed in senescent cells and furthermore, it remains to be seen what the repercussions are for growth factor RTK/receptor localisation, activity or turnover in senescent cells with hypopolarised plasma membranes. Restoring membrane potential does not promote elongation of cilia to the length seen in control cells 14, indicating that while membrane potential is important there may be other mechanisms controlling PC formation, possibly including p16 expression and autophagy defects (see specific sections elsewhere in this review).

One further consideration regarding membrane potential and senescence is whether only the plasma membrane is affected. Changes in membrane potential of the mitochondria, for example, are intimately linked to its function and mitochondria have indeed been shown to become depolarised in senescence 69. At least part of the mechanism underlying mitochondrial membrane depolarisation is the increased ROS production by dysfunctional mitochondria 69. These mitochondrial phenotypes are the key mediators of ageing and their dysfunction contributes to senescence acquisition and maintenance. It will be interesting to investigate the relationship between plasma membrane and mitochondrial membrane depolarisation. One intriguing possibility is that mitochondrial dysfunction results in the reduced levels of NADH which is required for the maintenance of plasma membrane potential.

It is not clear at the moment why the membrane potential of senescent cells is reduced, whether it is associated with de‐differentiation phenotypes, a direct result of increased cell size, defects in specific protein expression or changes in intracellular NADH and ROS balance. Furthermore, hypopolarisation of the cell membrane would impact intracellular calcium levels, which can directly influence growth factor signalling and AMPK activity and it will be interesting to explore how these changes, as well as the balance of other ions affect signalling in senescent cells.

## Autophagy and cilia

In addition to the role in growth factor signal transduction, PC have a reciprocal regulatory relationship with autophagy albeit one that is not completely clear at present 56, 70, 71. Autophagy, both basal and starvation‐induced, can promote the degradation of specific ciliary proteins including IFT20 and the negative regulator OFD1. As a result, autophagy has been shown to both positively and negatively affect PC growth 70, 71; indeed, autophagy perturbation in the form of *Atg5*/*Atg7* KO has been shown to both increase 70 and reduce 56, 71 PC formation and length. An explanation for these differences remains elusive but may perhaps be due to differences in cell type or in response to specific stimulus. In the opposite direction, PC and Hh signalling have been shown to contribute to control of autophagosome formation from the plasma membrane 70 although similarly, other reports suggest Hh signalling negatively regulates autophagy 72. Further evidence suggests autophagy may also participate in the cilia‐LKB1‐AMPK‐mTOR pathway in controlling fluid flow‐dependent cell size in the kidney 55, 73. One further consideration is that autophagy may regulate the turnover of other PC proteins and that the senescence‐associated perturbation of autophagy could impact on PC formation, turnover and/or the subsequent recruitment of proteins controlling growth factor signalling.

## mTORC1 responsiveness to other mitogenic signals in senescence

While we and others have shown that mTORC1 and autophagy are less sensitive to changes in nutrients, it is not clear at present whether mTORC1 activity responds properly to other stimuli. For example, oxidative stress is a common hallmark of all forms of senescence 74 and indeed exogenous oxidative stress, for example, prolonged exposure to H_2_O_2_ 75 can promote senescence induction while hypoxia can suppress senescence onset 74. The translation and subsequent activity of proteins important for controlling redox balance such as HIF1a are under control of mTORC1 and therefore changes in mTORC1 responsiveness to mitogenic cues may impact redox balance 76. In the reciprocal relationship, ROS has been demonstrated to be able to both activate and inhibit mTORC1 depending on duration and concentration. Age‐related mitochondrial stress can promote the ROS‐dependent activation of mTORC1 and induction of senescence 77 and at the same time, increased mitochondrial content in senescent cells is associated with increased ROS which activates DDR signalling pathway to control mTORC1 30. So while there are clear links between oxidative stress and senescence, the potential specific role and mechanisms via which mTORC1 may control this cross‐talk requires more work.

The activity of mTORC1 is also controlled by energy availability which is transduced via AMPK upon its activation by increased intracellular AMP/ADP levels (i.e. a drop in ATP) to TSC complex and Rheb activity. Reports have shown that AMPK activity is reduced in senescence and its activation may suppress oxidative stress‐induced senescence 42; this correlates with the fact that AMPK responsiveness reduces with age and its activation can increase lifespan 78. Other reports, however, suggest AMPK is activated in senescence due to elevated AMP:ATP ratio which has been associated with reduced mitochondrial function and therefore ATP production and that AMPK activation can drive senescence 79, 80, 81 including via senescence‐associated elevation of LKB1 82. We were unable to detect any gross changes AMPK phosphorylation at steady state in senescence human lung fibroblasts, but our insights into nutrient sensing by mTORC1 suggest that responsiveness to energy levels (or Ca^2+^) may still be perturbed. Equally, we observed that TSC2 is not recruited robustly to the lysosome in starved senescent cells which supports mTORC1 activity and although we attributed this to persistent PI3K/Akt signalling, we cannot rule out that the responsiveness or activity of TSC2 to AMPK signalling is disrupted in senescence 14.

## Implications for ageing

Ageing and age‐related diseases such as neurodegeneration and cancer are some of the most costly burdens to our society today, both economically and socially. Identifying interventions that may support healthy ageing is one of the most important questions of our time. Excitingly, the clearance of senescent cells has recently been established as a powerful approach to promote both health‐ and life‐span extension in mice 83. Thus, it has been demonstrated that removal of senescent cells can reverse age‐related functional decline in the heart, liver, bone, lung and prevent development of a wide range of diseases including atherosclerosis and diabetes mellitus. These findings have important potential implications for anti‐ageing interventions in humans. Indeed, human trials are underway where senolytics will be tested for their beneficial effect in age‐related diseases, multimorbidity and frailty 83.

We have shown that correcting the phenotypes in the nutrient sensing pathways of senescent cells can promote selective senescent cell death *in vitro* 14. As such, our mechanistic investigations have added a number of new drugs such as those targeting plasma membrane polarisation (pinacidil), Akt and mTORC1 inhibitors (such as rapamycin and torin 1) and autophagy blockers (chloroquine) to the growing list of potential senolytics. Some of these interventions, such as autophagy inhibition have already been demonstrated to kill senescent cells using *in vivo* models of anticancer chemotherapy‐induced senescence 84. Many other interventions such as rapamycin, caloric restriction and exercise that have most consistent and potent ability to extend health‐ and lifespan directly influence mTORC1 and autophagy. It would therefore be interesting to investigate whether any of the previously observed beneficial effects of these interventions are mediated by their senolytic properties *in vivo*.

## Conclusion

The realisation that senescent cells contribute to age‐related tissue decline has made the identification of senolytic compounds an area of particular importance to biomedicine. The acquisition and maintenance of cellular senescence is associated with fundamental rewiring of transcription, translation and metabolism and a better understanding of these changes will undoubtedly help support this quest to find healthy ageing interventions.

In this review, we have discussed how the mTORC1‐autophagy signalling axis becomes unresponsive to growth factors and amino acids in senescence and that how this may support the characteristic pro‐growth phenotypes. The true contribution of phenotypes we have discussed here such as elevated basal autophagy, membrane hypopolarisation and PC loss to senescence remain important future directions for the field. Future work will also help to unravel which of the extensive senescence‐associated changes reported are causal mechanisms or consequences. For example, as we have discussed, mTORC1 clearly regulates senescence‐associated phenotypes such as SASP; however, rapamycin has never been demonstrated to rescue cell cycle arrest. Thus, additional mechanisms upstream or parallel to mTORC1 clearly control this aspect of senescence.
